# Supplementary material for: HIVprotI: an integrated web based platform for prediction and design of HIV proteins inhibitors
Source: J Cheminform. 2018 Mar 9;10:12. doi: 10.1186/s13321-018-0266-y (PMC5845081; doi:10.1186/s13321-018-0266-y)
Supplement: Supplementary file 2 — Additional file 2. Source code of HIVProtI web server. [file 13321_2018_266_MOESM2_ESM.zip › HIVprotI_Source-code/hivprotI/weka-3-6-6/doc/weka/filters/supervised/attribute/AttributeSelection.html]

AttributeSelection


---


|  |  |  |
| --- | --- | --- |
| **Overview** \| **Package** \| **Class** \| **Tree** \| **Deprecated** \| **Index** \| **Help** \| **Weka's home** | |  |
| **PREV CLASS**   **NEXT CLASS** | **FRAMES**    **NO FRAMES**     **All Classes** |  |
| SUMMARY: NESTED \| FIELD \| CONSTR \| METHOD | DETAIL: FIELD \| CONSTR \| METHOD |  |


---


## weka.filters.supervised.attribute Class AttributeSelection

```
java.lang.Object
  weka.filters.Filter
      weka.filters.supervised.attribute.AttributeSelection
```

**All Implemented Interfaces:**: java.io.Serializable, CapabilitiesHandler, OptionHandler, RevisionHandler, SupervisedFilter

---

``` public class AttributeSelection extends Filter implements SupervisedFilter, OptionHandler ```

A supervised attribute filter that can be used to select attributes. It is very flexible and allows various search and evaluation methods to be combined.


Valid options are:

```
 -S <"Name of search class [search options]">
  Sets search method for subset evaluators.
  eg. -S "weka.attributeSelection.BestFirst -S 8"
```

```
 -E <"Name of attribute/subset evaluation class [evaluator options]">
  Sets attribute/subset evaluator.
  eg. -E "weka.attributeSelection.CfsSubsetEval -L"
```

```
 Options specific to evaluator weka.attributeSelection.CfsSubsetEval:
```

```
 -M
  Treat missing values as a seperate value.
```

```
 -L
  Don't include locally predictive attributes.
```

```
 Options specific to search weka.attributeSelection.BestFirst:
```

```
 -P <start set>
  Specify a starting set of attributes.
  Eg. 1,3,5-7.
```

```
 -D <0 = backward | 1 = forward | 2 = bi-directional>
  Direction of search. (default = 1).
```

```
 -N <num>
  Number of non-improving nodes to
  consider before terminating search.
```

```
 -S <num>
  Size of lookup cache for evaluated subsets.
  Expressed as a multiple of the number of
  attributes in the data set. (default = 1)
```

**Version:**
:   $Revision: 5541 $

**Author:**
:   Mark Hall (mhall@cs.waikato.ac.nz)

**See Also:**: Serialized Form

---

**Constructor Summary**

`AttributeSelection()`
  
          Constructor


| **Method Summary** | |
| --- | --- |
| `boolean` | `batchFinished()`             Signify that this batch of input to the filter is finished. |
| `java.lang.String` | `evaluatorTipText()`             Returns the tip text for this property |
| `Capabilities` | `getCapabilities()`             Returns the Capabilities of this filter. |
| `ASEvaluation` | `getEvaluator()`             Get the name of the attribute/subset evaluator |
| `java.lang.String[]` | `getOptions()`             Gets the current settings for the attribute selection (search, evaluator) etc. |
| `java.lang.String` | `getRevision()`             Returns the revision string. |
| `ASSearch` | `getSearch()`             Get the name of the search method |
| `java.lang.String` | `globalInfo()`             Returns a string describing this filter |
| `boolean` | `input(Instance instance)`             Input an instance for filtering. |
| `java.util.Enumeration` | `listOptions()`             Returns an enumeration describing the available options. |
| `static void` | `main(java.lang.String[] argv)`             Main method for testing this class. |
| `java.lang.String` | `searchTipText()`             Returns the tip text for this property |
| `void` | `setEvaluator(ASEvaluation evaluator)`             set attribute/subset evaluator |
| `void` | `setOptions(java.lang.String[] options)`             Parses a given list of options. |
| `void` | `setSearch(ASSearch search)`             Set search class |

**Methods inherited from class weka.filters.Filter**

`batchFilterFile, filterFile, getCapabilities, getOutputFormat, isFirstBatchDone, isNewBatch, isOutputFormatDefined, makeCopies, makeCopy, numPendingOutput, output, outputPeek, setInputFormat, toString, useFilter, wekaStaticWrapper`

**Methods inherited from class java.lang.Object**

`equals, getClass, hashCode, notify, notifyAll, wait, wait, wait`

**Constructor Detail**

### AttributeSelection

```
public AttributeSelection()
```

:   Constructor


**Method Detail**

### globalInfo

```
public java.lang.String globalInfo()
```

:   Returns a string describing this filter

    :   **Returns:**: a description of the filter suitable for displaying in the explorer/experimenter gui

---


### listOptions

```
public java.util.Enumeration listOptions()
```

:   Returns an enumeration describing the available options.

    :   **Specified by:**: `listOptions` in interface `OptionHandler`
    :   **Returns:**: an enumeration of all the available options.

---


### setOptions

```
public void setOptions(java.lang.String[] options)
                throws java.lang.Exception
```

:   Parses a given list of options.


    Valid options are:

    ```
     -S <"Name of search class [search options]">
      Sets search method for subset evaluators.
      eg. -S "weka.attributeSelection.BestFirst -S 8"
    ```

    ```
     -E <"Name of attribute/subset evaluation class [evaluator options]">
      Sets attribute/subset evaluator.
      eg. -E "weka.attributeSelection.CfsSubsetEval -L"
    ```

    ```
     Options specific to evaluator weka.attributeSelection.CfsSubsetEval:
    ```

    ```
     -M
      Treat missing values as a seperate value.
    ```

    ```
     -L
      Don't include locally predictive attributes.
    ```

    ```
     Options specific to search weka.attributeSelection.BestFirst:
    ```

    ```
     -P <start set>
      Specify a starting set of attributes.
      Eg. 1,3,5-7.
    ```

    ```
     -D <0 = backward | 1 = forward | 2 = bi-directional>
      Direction of search. (default = 1).
    ```

    ```
     -N <num>
      Number of non-improving nodes to
      consider before terminating search.
    ```

    ```
     -S <num>
      Size of lookup cache for evaluated subsets.
      Expressed as a multiple of the number of
      attributes in the data set. (default = 1)
    ```

    :   **Specified by:**: `setOptions` in interface `OptionHandler`
    :   **Parameters:**: `options` - the list of options as an array of strings **Throws:**: `java.lang.Exception` - if an option is not supported

---


### getOptions

```
public java.lang.String[] getOptions()
```

:   Gets the current settings for the attribute selection (search, evaluator)
    etc.

    :   **Specified by:**: `getOptions` in interface `OptionHandler`
    :   **Returns:**: an array of strings suitable for passing to setOptions()

---


### evaluatorTipText

```
public java.lang.String evaluatorTipText()
```

:   Returns the tip text for this property

    :   **Returns:**: tip text for this property suitable for displaying in the explorer/experimenter gui

---


### setEvaluator

```
public void setEvaluator(ASEvaluation evaluator)
```

:   set attribute/subset evaluator

    :   **Parameters:**: `evaluator` - the evaluator to use

---


### searchTipText

```
public java.lang.String searchTipText()
```

:   Returns the tip text for this property

    :   **Returns:**: tip text for this property suitable for displaying in the explorer/experimenter gui

---


### setSearch

```
public void setSearch(ASSearch search)
```

:   Set search class

    :   **Parameters:**: `search` - the search class to use

---


### getEvaluator

```
public ASEvaluation getEvaluator()
```

:   Get the name of the attribute/subset evaluator

    :   **Returns:**: the name of the attribute/subset evaluator as a string

---


### getSearch

```
public ASSearch getSearch()
```

:   Get the name of the search method

    :   **Returns:**: the name of the search method as a string

---


### getCapabilities

```
public Capabilities getCapabilities()
```

:   Returns the Capabilities of this filter.

    :   **Specified by:**: `getCapabilities` in interface `CapabilitiesHandler` **Overrides:**: `getCapabilities` in class `Filter`
    :   **Returns:**: the capabilities of this object **See Also:**: `Capabilities`

---


### input

```
public boolean input(Instance instance)
              throws java.lang.Exception
```

:   Input an instance for filtering. Ordinarily the instance is processed
    and made available for output immediately. Some filters require all
    instances be read before producing output.

    :   **Overrides:**: `input` in class `Filter`
    :   **Parameters:**: `instance` - the input instance **Returns:**: true if the filtered instance may now be collected with output(). **Throws:**: `java.lang.IllegalStateException` - if no input format has been defined.: `java.lang.Exception` - if the input instance was not of the correct format or if there was a problem with the filtering.

---


### batchFinished

```
public boolean batchFinished()
                      throws java.lang.Exception
```

:   Signify that this batch of input to the filter is finished. If the filter
    requires all instances prior to filtering, output() may now be called
    to retrieve the filtered instances.

    :   **Overrides:**: `batchFinished` in class `Filter`
    :   **Returns:**: true if there are instances pending output. **Throws:**: `java.lang.IllegalStateException` - if no input structure has been defined.: `java.lang.Exception` - if there is a problem during the attribute selection.

---


### getRevision

```
public java.lang.String getRevision()
```

:   Returns the revision string.

    :   **Specified by:**: `getRevision` in interface `RevisionHandler` **Overrides:**: `getRevision` in class `Filter`
    :   **Returns:**: the revision

---


### main

```
public static void main(java.lang.String[] argv)
```

:   Main method for testing this class.

    :   **Parameters:**: `argv` - should contain arguments to the filter: use -h for help


---


|  |  |  |
| --- | --- | --- |
| **Overview** \| **Package** \| **Class** \| **Tree** \| **Deprecated** \| **Index** \| **Help** \| **Weka's home** | |  |
| **PREV CLASS**   **NEXT CLASS** | **FRAMES**    **NO FRAMES**     **All Classes** |  |
| SUMMARY: NESTED \| FIELD \| CONSTR \| METHOD | DETAIL: FIELD \| CONSTR \| METHOD |  |


---
